# Polybenzimidazole Membranes Doped with Sulfonic Acid-Containing Covalent Organic Frameworks and Polymers for Enhanced Performance in Vanadium Redox Flow Batteries

**DOI:** 10.1021/acsami.5c00568

**Published:** 2025-06-23

**Authors:** Beyadgalem Endawoke Anley, Cheng-Ju Yu, Tsung-Yun Wu, Chun-Chiang Huang, Jun-Sheng Wang, Hsieh-Chih Tsai

**Affiliations:** † Graduate Institutes of Applied Science and Technology, 34878 National Taiwan University of Science and Technology, Taipei 106, Taiwan, Republic of China; ‡ Advanced Membrane Materials Center, National Taiwan University of Science and Technology, Taipei 106, Taiwan, Republic of China; § Taiwan Instrument Research Institute, 87786 National Applied Research Laboratories, Hsinchu 302, Taiwan, Republic of China; ∥ R&D Center for Membrane Technology, Chung Yuan Christian University, Chungli, Taoyuan 320, Taiwan, Republic of China

**Keywords:** polybenzimidazole (PBI), PBI composite membranes, vanadium redox flow battery (VRFB), efficiency, durability

## Abstract

Polybenzimidazole (PBI)-based membranes in vanadium redox flow batteries (VRFBs) achieve high Coulombic efficiency due to their excellent ion selectivity; however, their energy efficiency remains relatively low due to restricted proton transport. Covalent organic framework (COF) and polymer (COP)-based proton-selective membranes have been developed to address the trade-off between proton permeability and selectivity, significantly improving the redox flow battery performance, including all-vanadium systems. These membranes achieve precise proton and vanadium ion separation with an optimized pore size, reducing vanadium ion crossover while maintaining low area resistance through their microporous structure and imine group functionalities. Single electrochemical cells with these membranes demonstrate high Coulombic efficiencies (94.18–99.19%) and energy efficiencies (75.37–88.50%) at current densities of 40–100 mA cm^–2^. The integration of PBI with the sulfonic acid-bearing COFs and COPs enhances the membranes’ electrochemical performance as well as their chemical stability, ensuring long-term cycle durability. This work presents a promising approach for designing ultrahigh-proton-selective membranes, offering valuable insights for advanced redox flow batteries and other electrochemical systems.

## Graphical abstract

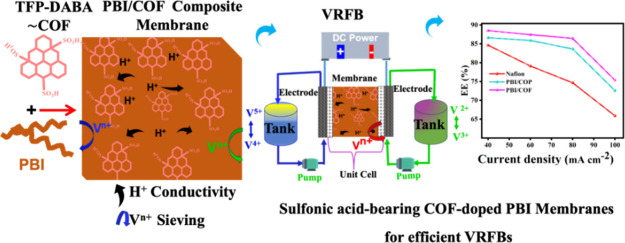

## 1 Introduction

The need for decarbonization and the demand for sustainable, renewable energy are more critical than ever.^1,2^ The intermittent nature of these renewable energy sources creates a mismatch between supply and demand due to environmental conditions, highlighting the need for efficient, large-scale energy storage systems.^3,4^ These energy storage systems store surplus energy and release it when needed, ensuring grid stability.^5^ Batteries, particularly vanadium redox flow batteries (VRFBs), have gained prominence due to their scalability and ability to store excess energy on demand. VRFBs offer distinct advantages such as long cycle life, flexible operation, and decoupled power and energy capacity, making them ideal for managing energy variability in renewable energy grids. VRFBs have gained attention as a leading solution for large-scale, long-duration energy storage.^6,7^ Unlike solid-state batteries, VRFBs use vanadium ions in liquid electrolytes to store and release energy, allowing for scalable energy storage by adjusting the electrolyte volume without altering the power capacity. This flexibility makes VRFBs ideal for grid-scale applications, ensuring energy storage and grid stability.^8^ Their long lifespan, minimal degradation, and nonflammable, aqueous electrolytes further enhance safety and reliability, making them pivotal for renewable energy integration and decarbonization efforts.^9^ Unlike conventional batteries, which degrade over time, VRFBs use vanadium ions in different oxidation states within liquid electrolytes.^10^ This allows them to be recycled indefinitely with minimal capacity loss, reducing the need for frequent replacement and hazardous waste generation.^11^ Vanadium’s reusability further minimizes its environmental footprint, making it a greener alternative to technologies like lithium-ion batteries. They are also highly scalable because increasing the energy capacity is as simple as enlarging the electrolyte tanks, making them ideal for grid stabilization and renewable energy integration.^12^ However, a key challenge lies in the ion-exchange membrane performance. These membranes must balance high proton conductivity with low vanadium ion crossover to maintain efficiency and battery life.^9,13^ Nafion, a commonly used membrane, suffers from high vanadium permeability, leading to self-discharge and capacity fade. Moreover, environmental concerns surrounding perfluorinated compounds in Nafion have driven the search for alternatives.^14^ Polybenzimidazole (PBI) has emerged as a promising candidate due to its chemical stability and reduced vanadium ion crossover when doped with acids.^15^

PBI is a high-performance polymer known for its outstanding thermal and chemical stability. However, it lacks conductivity in its pure form. When exposed to acidic solutions, PBI becomes protonated, absorbing acid and water to enable proton conduction.^16^ Its narrow, positively charged channels in sulfuric acid-doped PBI effectively block vanadium ion crossover through size exclusion, making it a strong candidate for VRFB membranes.^17^ Membranes cast from organic solutions of pristine PBI exhibited low ionic conductivity and high area-specific resistance at practical thicknesses, limiting their energy efficiency and current density.^18^ While methods like grafting bulky side chains or preswelling in acids or bases can enhance ion transport, they frequently lead to increased vanadium crossover and reduced long-term stability.^19^ To overcome this challenge, designing well-ordered, porous-structured membrane materials that selectively facilitate ion transport while effectively restricting vanadium ion permeation through the pore walls emerges as a compelling strategy.^20^ A more sustainable solution involves incorporating ionic-moiety-containing porous materials into the polymer backbone to improve the selectivity of the VRFB membrane.^21^ Furthermore, integrating covalent organic frameworks (COFs) and polymers (COPs) with PBI membranes has shown to effectively improve the performance.^22^ In this context, COFs and COPs have emerged as advanced materials for energy storage applications due to their tunable architecture, robust covalent bonding, and exceptional stability.^23,24^ COFs, characterized by their ordered reticular frameworks and high crystallinity, enable rapid ion transport through well-defined porous networks, enhancing charge storage and cycling stability.^25^ Meanwhile, COPs, with their amorphous yet processable structures, provide mechanical adaptability and chemical versatility, optimizing the electrochemical performance.^26^ COFs and COPs are promising materials for energy storage and conversion due to their high surface area and tunable porosity and the presence of redox-active sites. However, their practical application is often limited by high synthesis costs, restricted solubility, and poor processability.^27^ To address these challenges, we developed a scalable and cost-effective interfacial thermal condensation polymerization (IP) method for synthesizing porous free-standing networks based on 1,3,5-triformylphloroglucinol (TFP) and 2,5-diaminobenzene-1,4-disulfonic acid (DABA) as a precursor employing different solvent systems. The combinations of mesitylene/dioxane yields reversible TFP–DABA COFs, while toluene/dioxane tends to yield irreversible TFP–DABA COP networks, with the same precursor yielding different porosities due to variations in the solvent diffusivity, interfacial tension, and kinetic control over the network growth. The IP of TFP–DABA occurs at the solvent interface, where the interplay between the solvent diffusivity and interfacial tension governs the network growth. Thus, solvent diffusivity facilitates the controlled transport of monomers to the interface, ensuring homogeneous mixing and promoting kinetically driven imine condensation.^28^ This approach stabilizes the dynamic thermal polycondensation process and promotes the formation of ultrathin, free-standing networks. The resulting frameworks, which contain both imine and sulfonic acid functional groups, demonstrate an enhanced compatibility with the imidazole domains of PBI chains. This in situ growth approach minimizes aggregation and promotes intimate interfacial contact with the polymer phase, resulting in homogeneous COF/COP dispersion within the PBI matrix. This facilitates the molecular-level dispersion of monomers, preventing premature precipitation.^29^ Our setup establishes a foundation for uniform nucleation and interfacial growth, enabling early-stage integration of these materials into dense, defect-free membranes. The optimized membrane fabrication protocol, which combines ultrasonication and controlled solvent evaporation, further ensures that the resulting films are free-standing, uniform, and mechanically stable. These films exhibit improved mechanical integrity and enhanced ion-transport properties, thereby advancing their applicability in VRFB systems.

In this work, we address the persistent trade-off between proton conductivity and vanadium ion selectivity in PBI-based membranes for VRFBs by incorporating sulfonic acid-functionalized covalent organic frameworks (sCOFs) and polymers (sCOPs) into the PBI matrix. We establish a strategy that enables molecular-level control over the membrane porosity, ionic functionality, and structural integrity. The sulfonic acid moieties serve dual roles: they protonate the benzimidazole units of PBI, enhancing proton transport, while simultaneously mitigating vanadium ion crossover via hydrogen bonding, size exclusion, and electrostatic repulsion.^30^ Unlike conventional sulfonated PBI membranes, which suffer from vanadium permeability despite high conductivity, our sCOF/PBI and sCOP/PBI composites are engineered to introduce well-defined ionic channels with tailored connectivity and stability. This approach not only improves proton conductivity but also imparts superior mechanical robustness and electrochemical selectivity.^31^ Here, we systematically evaluate the membrane fabrication and operating conditions. The well-defined and highly performing PBI composite membranes demonstrate significantly higher proton conductivity than PBI membranes free of sulfonic acid-bearing composites. The incorporation of sulfonic acid-containing sCOF and sCOP introduces additional ionic pathways for proton transport, enhancing both the membrane’s conductivity and its overall integrity. The resulting membranes represent a promising platform for advancing the VRFB performance through a tunable and scalable material design.

## 2 Experimental Section

### 2.1 Materials

Polybenzimidazole (PBI, Microcosm), 1,3,5-triformylphloroglucinol (TPF, 98%), 2,5-diaminobenzene-1,4-disulfonic acid (DABA, 97%), tetrahydrofuran (THF, 98%), dimethylformamide (DMF), and *N*-methyl-2-pyrrolidone (NMP, 97%) were purchased from Sigma-Aldrich and used as received. Anhydrous dichloromethane (CH_2_Cl_2_), toluene (97%), and 1,4-dioxane (98%) were obtained from Thermo-Scientific and used without further purification. A Nafion dispersion of 1-propanol (∼50%), water (∼38%), ethyl alcohol (<5%), and others in a specified weight percent was used as a standard for comparison as “Nafion” throughout the paper; all reagents and chemicals used in this study were of analytical grade, and deionized water, obtained from our laboratory, was used throughout the experiment. COF and COP were synthesized in our laboratory based on concepts from recent studies^32^ and using the protocols outlined in Scheme 1.

**Scheme 1:**
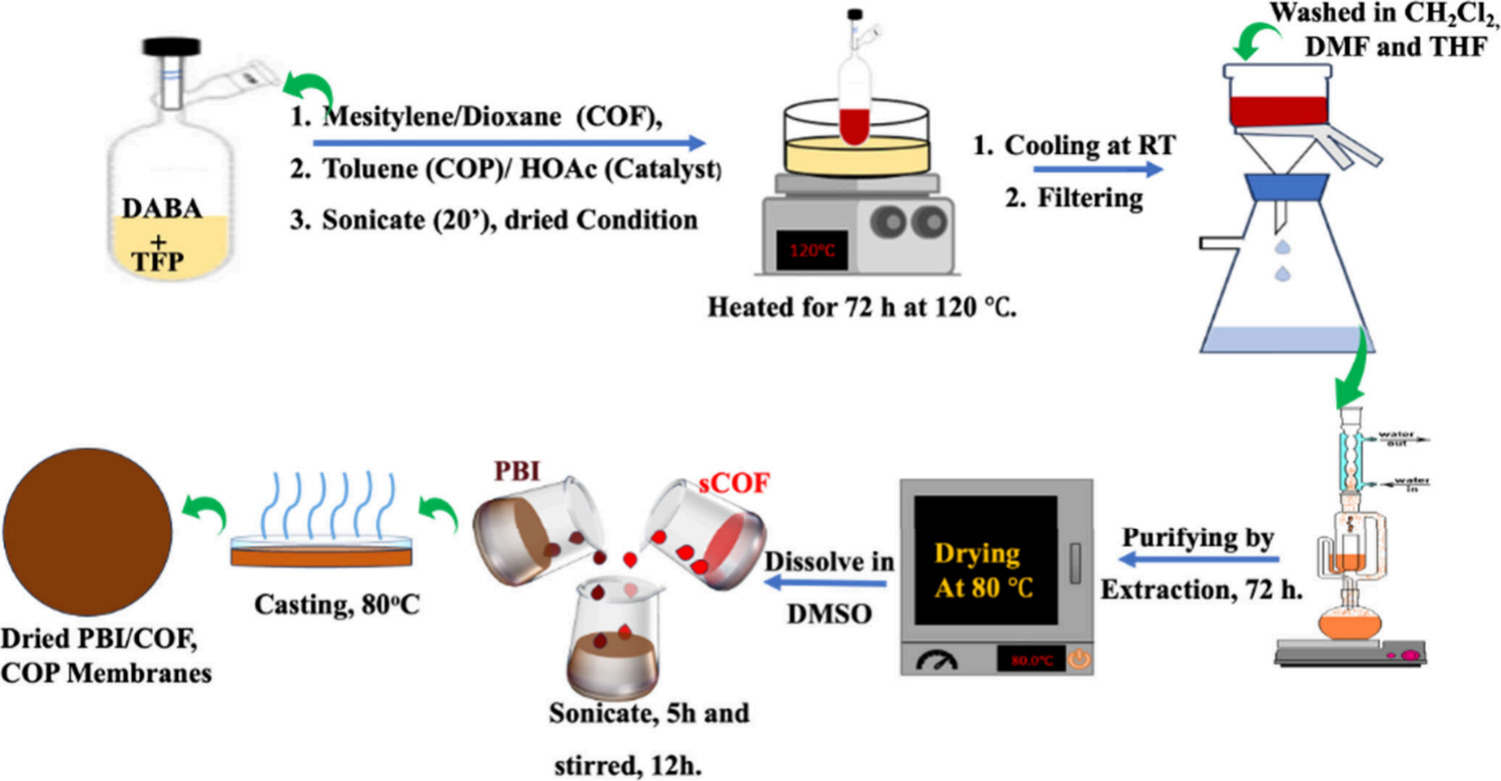
Schematic Representation of the Synthesis Routes for COF, COP, and PBI Composite Membranes

### 2.2 Synthesis of Sulfonic Acid-Containing COFs and COPs

Finely ground samples of DABA (28.2 mg, 0.15 mmol), TFP (21.0 mg, 0.1 mmol), mesitylene (3 mL), dioxane (1 mL), and acetic acid (6M, 0.5 mL) were added to the reactor and oscillated with ultrasonic waves for 20 min, followed by sonication, and dissolved oxygen gas was removed through freeze–pump–thaw cycles. The solution was then heated to 120 °C for 72 h. Upon completion, the reaction mixture was cooled to room temperature, and the resulting product was filtered via suction filtration. The obtained powder was thoroughly washed with acetone, CH_2_Cl_2_, THF, and DMF and then purified using Soxhlet extraction for 72 h using anhydrous methanol and THF. The sample was dried in a vacuum oven at 80 °C for 24 h, yielding a dark-red sample of sCOF. The dried material, identified as sCOF based on the starting material, and sCOP was also synthesized using toluene as the precursor solvent while keeping all other conditions unchanged.

### 2.3 Preparations of COF, COP, and PBI Composite Membranes

A total of 7 mg of COF was dispersed in 7 mL of NMP and sonicated for 20 min to reduce the particle size. The dispersion was then centrifuged at 3000 rpm for 10 min, and the supernatant was collected to achieve uniform distribution. Afterward, predetermined amounts of PBI were added to the COF dispersions and stirred magnetically for 24 h to ensure complete mixing. The resulting solution was cast onto a Teflon crystallizing plate (4 cm diameter) and heated at 80 °C to evaporate the solvent, producing a bright-yellowish PBI/COF composite film, which was obtained after careful peeling from the Teflon plate and conditioning for characterizations. The PBI/COP composite films were also prepared in the same way.

### 2.4 Characterizations of the Composite Membranes

The integration of sulfonic acid-containing COF and COP into the PBI membrane was confirmed through ^13^C NMR analysis, using an Agilent 600-MR instrument (600 MHz) with CDCl_3_ as the solvent. Moreover, the vibrational transitions were verified by Fourier transform infrared (FT-IR)-ATR spectroscopy within the range of 4000–600 cm^–1^ in attenuated total reflection mode, with a scan rate of 32 s and resolutions of 4. The surface and cross-sectional micromorphology of the PBI/sCOF/COP composite membranes were characterized by field-emission scanning electron microscopy (SEM, JSM 6000F, JEOL, Tokyo, Japan); furthermore, the nanoscale crystallinity of the nanosheet formed between the interface was analyzed by employing a X-ray diffractometer (Bruker, D2PHASED-XRD) with Cu Kα radiations at a scan speed of 5° min^–1^ within the range of 3–30° (2θ) as the function’s Bragg’s law of diffraction.

### 2.5 Ion-Exchange Capacity (IEC), Water Uptake (*W*_U_), and Swelling Ratio (*S*_R_)

The IECs of AEM’s PBI composite membranes were determined after the soaking of a specified well-dried gram of a membrane sample in 1 M H_2_SO_4_ for 24 h. Upon washing with deionized water, the sample was resoaked in a 0.01 M NaCl solution for 24 h, and the resulting solution was titrated with a standard 0.01 M NaCl solution. Finally, the IEC values were determined via eq 1.

1 $$\mathrm{IEC}=\frac{C\left(V_{1}-V_{2}\right)}{M_{\mathrm{dry}}}$$

*C* is the concentration of a standard NaOH solution (mol L^–1^), *V*_1_ and *V*_2_ are the volumes (mL) of NaCl used to immerse the membrane and NaOH used for the titrations of residual NaCl over the dried membrane, and *M*_dry_ stands for the dry weight of the membrane. To determine *W*_U_ and *S*_R_ of the PBI composite membranes, a 1 cm × 4 cm size of predried membranes was prepared, and the weight was recorded. Afterward, the samples were immersed in deionized water at room temperatures for 72 h. *W*_U_ and *S*_R_ of the membranes were calculated using eqs 2 and 3.

2 $$\mathrm{water}\;\mathrm{uptake}=\frac{M_{\mathrm{wet}}-M_{\mathrm{dry}}}{M_{\mathrm{dry}}}\times 100$$

3 $$\mathrm{swelling ratio}=\frac{\mathrm{wet membrane surface area}-\mathrm{dry membrane surface area}}{\mathrm{dry membrane surface area}}\times 100$$

*M*_wet_ and *M*_dry_ are the weights of the acid-doped PBI membranes and the vacuum-dried pristine PBI membranes.

### 2.6 Thermal and Acidic Stability of PBI Composite Membranes

The thermal stability of the thoroughly dried PBI composite membranes was evaluated using thermogravimetric analysis (TGA) on a Netzsch TGA/1600LF system with a temperature range of 30–900 °C at a heating rate of 10 °C min^–1^ under an inert atmosphere. The ex situ chemical stability of the membranes was tested by immersion in 0.1 M VO_2_^+^/3 M H_2_SO_4_ at 30 °C for 200 h. Changes in the membranes were monitored through physical observations of the surface conditions and by measurement of the conductivity after exposure to the acidic environment.

### 2.7 Ionic Conductivity (σ) and Area Resistance (Ω cm^2^) of the Membranes

The proton ion (H^+^) conductivity of membranes was measured by the four probe–electrode method with an alternating-current impedance analyzer (Bio-Logic SP-200) and in the frequency range of 1 Hz to 100 kHz per second with 2 cm × 2 cm sized functionalized PBI composite membranes. The H^+^ conductivity of membranes was calculated using eq 4 (mS cm^–1^) followed by area resistance (Ω) evaluations using electrochemical impedance spectroscopy.

4 $$\delta \;\left({\mathrm{mS cm}}^{-1}\right)=\frac{d}{LWR}$$

where *d*, *L*, and *W* are the distance of the two electrodes (1 cm) and the thickness and width of the sample, respectively. *R* is the measured resistance. The area resistance (*R*_A_) of the membranes was measured by using an H-type conductivity cell, which was filled with a 1.5 M VOSO_4_ solution and a 3 M H_2_SO_4_ solution on both sides. *R*_A_ was calculated using eq 5 as follows:

5 $$R_{A}=A\left(R_{1}-R_{2}\right)$$

where *A* is the effective area of the membrane sample and *R*_1_ and *R*_2_ are the resistances measured after and before the membrane was loaded, respectively.

### 2.8 Vanadium Ion (VO^2+^) Permeability and Single-Cell Performance

The permeability of VO^2+^ was evaluated using an H-type diffusion cell consisting of two compartments separated by the membrane sample. The donor compartment (left side) was filled with 100 mL of a 1.5 M VOSO_4_ solution in 2 M H_2_SO_4_, while the receiving compartment (right side) contained 100 mL of a 1.5 M MgSO_4_ solution in 2 M H_2_SO_4_, ensuring equal ionic strength in both compartments, as shown in Figure 1B. To mitigate concentration polarization and maintain homogeneous mixing, both compartments were continuously stirred at identical rates. At predetermined intervals (every 12–24 h), 1 mL samples were withdrawn from the receiving compartment (right side) and analyzed for VO^2+^ ion concentration via UV–vis spectroscopy at 765 nm. After each analysis, the sampled solution was returned to the receiving compartment to preserve consistent volume, while an equivalent amount of solution was simultaneously removed from the donor compartment to maintain constant hydrostatic pressure throughout the experiment, and the proton permeability was also tested following the same protocol. The permeability coefficient (ρ, cm^2^ min^–1^) was calculated based on the progressive increase in the VO^2+^ concentration in the receiving compartment over time based on simplified eq 6.

VO^2+^ permeability formula:

6 $$\begin{array}{l} j_{A}=V_{B}\;\frac{dC_{B}}{dt}=A\frac{P}{L}\left(C_{A}-C_{B}\right)\\ C_{A0}V_{A}=C_{A}V_{A}+C_{B}V_{B}\\ \mathrm{Assume:}\;V_{A}\approx V_{B}\to C_{A0}=C_{A}+C_{B}\\ V_{B}\;\frac{dC_{B}}{dt}=A\frac{P}{L}\left(C_{A0}-2C_{B}\right)\\ \mathrm{Initial condition:}\;t=0,\;C_{B}=0\\ \to -\ln\left(1-\frac{2C_{B}\left(t\right)}{C_{A0}}\right)=2A\frac{P}{V_{B}L}\left(t-t_{0}\right)\\ \rho =\frac{LV_{R}}{AC_{D}}\;\frac{{dC}_{R}}{dt} \end{array}$$

where *j*_A_ = vanadium ion flux, *t* = time, *A* = film area, *L* = film thickness, *P* = permeability, and *C* = solution concentration, and the ion selectivity (*S*) can be determined by applying the formula $S=\frac{\sigma }{P}$ , where σ = conductivity and *P* = permeability, as simplified in eq 6.

**Figure 1:**
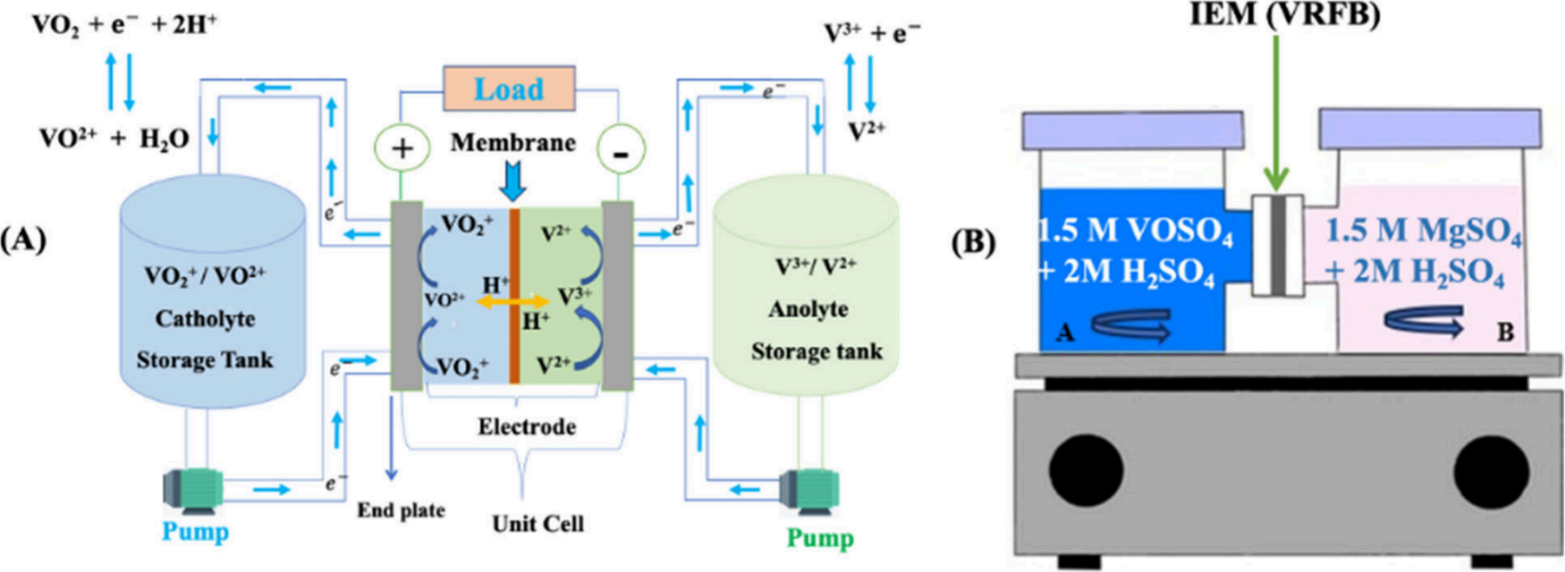
(A) VRFB working principle and (B) VO^2+^ permeability test.

The performance of VRFBs was evaluated using commercially available Nafion, PBI/sCOF, and sCOP composite membranes with an effective area of 4 × 4 cm^2^ that was placed between carbon felt electrodes (4 mm thick) in single-cell working stations (Figure 1A). The positive electrolyte consisted of 50 mL of 1.5 M VO^2+^/VO_2_^+^ in 3 M H_2_SO_4_, and the negative electrolyte was composed of 50 mL of V^2+^/V^3+^ in 3 M H_2_SO_4_. Charge–discharge cycles were performed at a 50 mV fixed voltage flow, with current densities ranging from 1 to 100 mA cm^–2^, using a LANHE battery tester (CT2001A). Nitrogen purging was applied to the electrolyte reservoirs to prevent V^2+^ oxidation prior to the performing cycle. Electrolyte activation was initiated by charging VO^2+^ at the anode, converting it to V^3+^, while VO_2_^+^ at the cathode was oxidized to V^5+^. Once V^5+^ was formed at the cathode, fresh VO^2+^ was introduced, continuing the charging process, converting V^3+^ to V^2+^ at the anode and V^4+^ to V^5+^ at the cathode, and ensuring consistent electrolyte activity throughout the test. The Coulomb efficiency (CE), voltage efficiency (VE), and energy efficiency (EE) under different current densities were calculated using eqs 7–9.

7 $$\mathrm{VE}\;\left(\%\right)=\frac{\int V_{D}\;dt}{\int V_{C}\;dt}\times 100\%$$

8 $$\mathrm{CE}\;\left(\%\right)=\frac{\int I_{D}\;dt}{\int I_{C}\;dt}\times 100\%$$

9 EE(%)=CE×VE×100%

where *V*_D_ is the discharge voltage, *V*_C_ is the charging voltage, *I*_D_ is the discharge current, and *I*_C_ is the charging current.

## 3 Results and Discussion

### 3.1 Chemical Structure Analysis

The selectivity, ion-transport efficiency, and durability of membranes for VRFB rest on the material properties and preparation methods. Herein, highly ordered, crystalline nanosheets of COFs and flexible, noncrystalline COPs were synthesized via interfacial thermal condensation polymerization in improved polymer growth at the interface of two immiscible solvents considering solvent effects over the combinations of DABA and TFP. The successful synthesis and material suitability were confirmed through comprehensive characterizations. As shown in Figure 2c, FT-IR spectroscopy in the ATR mode was employed to confirm the integrations between TFP and DABA before and after polymerization.^33^ The spectra exhibited bands at 1578 and 1238 cm^–1^, attributed to C=C and C–N stretching of the precursors, respectively. Notably, the C=O absorption at 1643 cm^–1^ from TFP and the −NH peaks at 3427 and 3336 cm^–1^ from DABA were absent in the spectra of the condensation products of COF and COP, confirming the successful imine (C=N) bond formations. Furthermore, peaks at 1026, 1080, and 1438 cm^–1^, corresponding to symmetric and asymmetric S=O stretches, indicated that the sulfonic acid groups from DABA remained intact. These spectral changes affirm the successful integration of TFP and DABA and the formation of a stable imine-linked polymer structure. Notably, COFs and COPs exhibit similar FT-IR vibrational and stretching peaks due to their shared precursor chemical compositions, differing mainly in crystallinity. Moreover, the surface elemental compositions of the free-standing ultrathin COFs and COPs and their PBI composite membranes were analyzed using deexcitation electron spectroscopy, as presented in Figures 2a,b and S1 and Table S1, respectively. The intensities and elemental ratios provided supplementary evidence supporting the FT-IR results. For instance, the sulfonic acid chain introduced sulfur, with mass ratios of 12% in COF and 7% in COP, thereby confirming the effective synthesis of the desired products. The incorporation of sulfonic acid-bearing porous COFs plays a dual role in enhancing both the structural integrity and functional performance of the membranes. The sulfonic acid chains within the networks not only facilitate hydrogen bonding but also significantly improve membrane hydration due to their pronounced hydrophilic properties. This increased hydration promotes the formation of well-defined ionic pathways, which are critical for the efficient proton hopping and sieving of vanadium ions. Furthermore, the sulfonic acid chains interact with the imine groups of the polymer backbone (PBI) to generate H^+^ ions, thereby boosting proton conductivity. This synergistic interaction between the functional groups enhances the formation of robust ionic channels, leading to an improved overall performance of the membrane.

**Figure 2:**
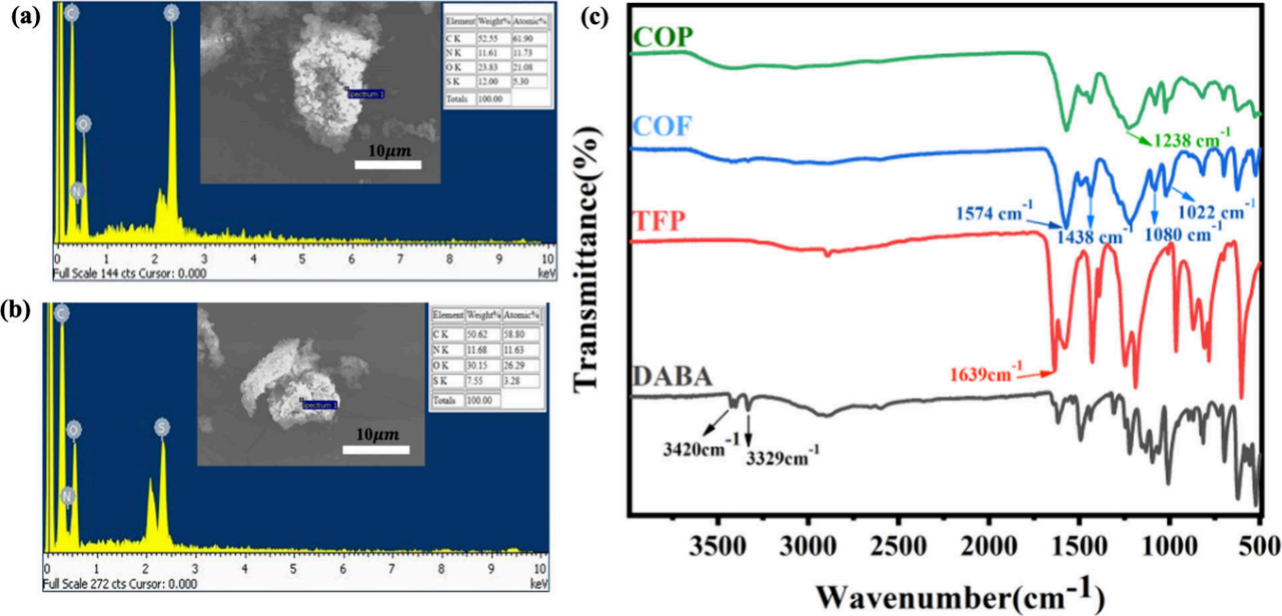
Energy-dispersive X-ray spectroscopy spectra of (a) sCOF and (b) sCOP and (c) FT-IR spectra of sCOF, sCOP, and the starting reagents in ATR mode.

The synthesis of COF and COP in their enol–keto forms was convincingly validated through ^13^C NMR spectroscopy, as presented in Figure 3b. The distinct signals near 180 ppm (182 ppm for COF and 181.2 ppm for COP) are strong indicators of the carbonyl carbons in the enol–keto form, reflecting the successful retention of this functional group during framework formation. These chemical shifts align well with the expected environment of conjugated enol carbons, further confirming the structural integrity of the frameworks. The emergence of imine (C–N) signals around 147 ppm signifies the successful condensation reaction between the aldehyde groups of TFP and the amine groups of DABA. The accompanying diminished signal due to consumption of the aldehyde carbonyl peak at 192 ppm underscores the reduction or nearly undergoes consumption of the starting aldehyde groups, marking the progression from monomers to a covalently bonded framework. Moreover, shifts in the aromatic carbon signals near 102 ppm highlight electronic reorganization within the conjugated system, providing further evidence of the framework’s structural transformation. Owing to their common precursors, the COFs and COPs display comparable FT-IR vibrational signatures and ^13^C NMR chemical environments, yet differ markedly in porosity and crystallinity, dictated by solvent diffusion and interfacial polymerization kinetics. These porosity differences critically influence the frameworks’ physicochemical profiles and functional applicability. These observations are critical because they highlight the adaptability of the synthetic strategy in producing frameworks with tailored porosity.^34^ The presence of sulfonic acid functional groups, while not directly observable in ^13^C NMR, plays an essential role in the framework’s properties. Their incorporation likely influences the electronic environment of nearby carbons, subtly contributing to the observed shifts. Altogether, the disappearance of starting material peaks or consumption, the emergence of imine peaks, and changes in the aromatic carbon regions collectively validate the successful synthesis of the desired frameworks and confirm the fidelity of the final structures. Moreover, X-ray diffraction (XRD) was also employed to investigate the crystalline structure of COF-SO_3_H and COP-SO_3_H, as shown in Figure 2c. The XRD patterns revealed two prominent peaks at 2θ = 4.7° and 27.62°, corresponding to the (100) and (001) planes, respectively. For COF-SO_3_H, a distinct peak at 4.7° was assigned to the (100) plane, indicating its orderly stacking. A slightly broader peak at 8.54° was attributed to the (110) plane, while another peak at 27.04° corresponded to the (001) plane as a result of π–π stacking.^33^ The alignment of the experimental peak positions confirms the successful synthesis and structural integrity of COF-SO_3_H and COP-SO_3_H. The sharp and intense peak of COF-SO_3_H at 4.7° reflects its superior crystallinity and higher surface area, whereas the absence of prominent peaks for COP-SO_3_H suggests an amorphous structure with less ordered packing and a lower surface area, which is attributed to the distinct chemistry and reaction conditions involved in their synthesis.

**Figure 3:**
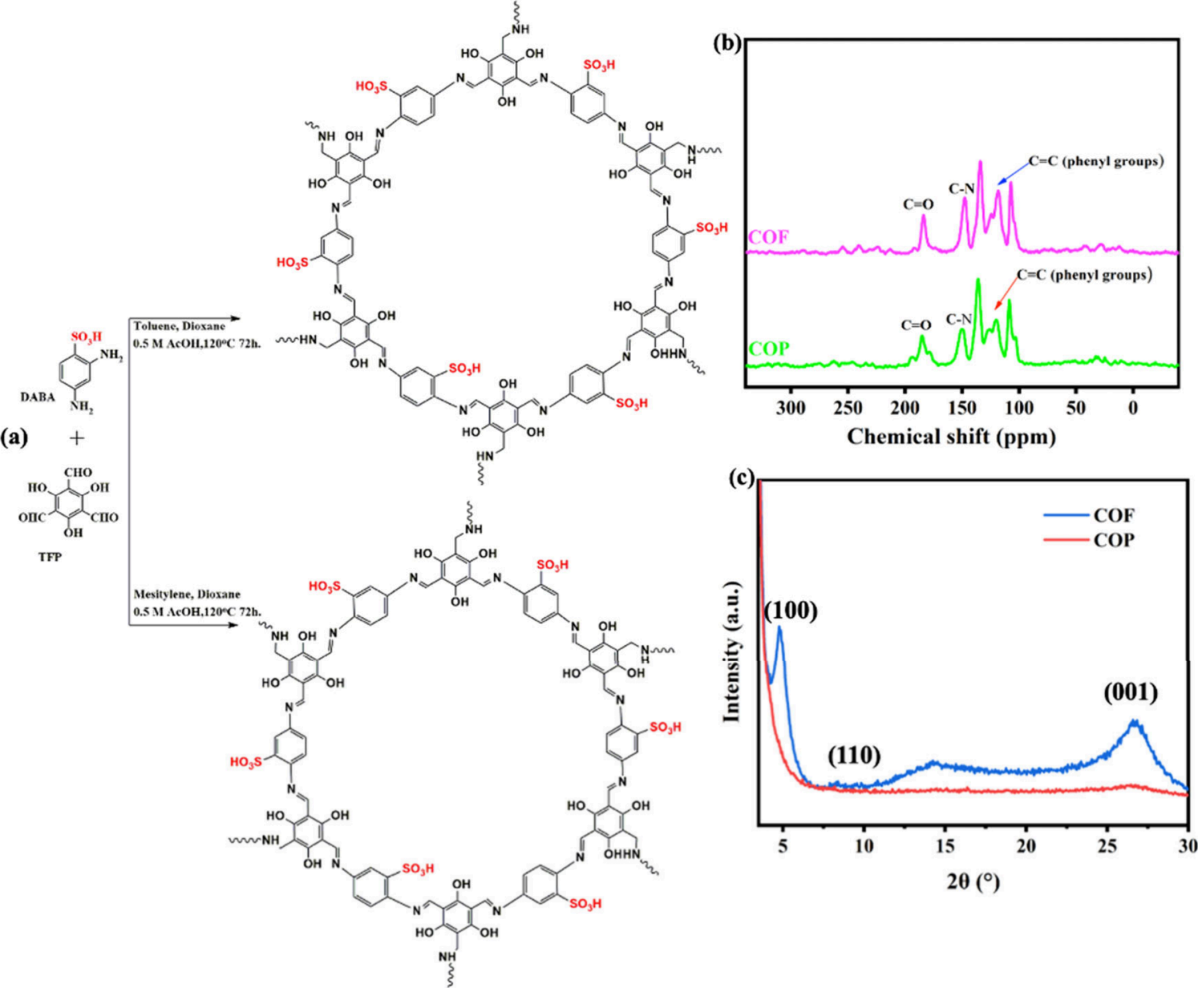
(a) Synthesis routes of sCOF and sCOP, (b) ^13^C NMR, and (c) XRD patterns of sCOF and sCOP.

### 3.2 Porosity and Surface Analysis of the Membranes

The N_2_ adsorption–desorption isotherms were utilized to assess the textural properties of the synthesized free-standing, ultrathin covalent organic networks, as presented in Figure S2. The Brunauer–Emmett–Teller (BET) surface areas of COF and COP were 110.99 and 29.42 m^2^ g^–1^, respectively, underscoring the enhanced porosity of the COF. This disparity can be primarily attributed to the highly ordered, crystalline nature of the COFs, which enables more efficient molecular packing and uniform pore distributions. In contrast, COPs possess an amorphous, lower-surface-area, cross-linked structure formed through random interconnections between precursors, leading to denser architectures and limited pore accessibility. Consistent with these surface area findings, the COF also displayed a greater pore volume of 0.191 cm^3^ g^–1^ than the COP of 0.109 cm^3^ g^–1^, further substantiating its superior porosity.^35^ The formation of COFs through the condensation of DABA and TFP results in a well-aligned, periodic framework with extended π–π stacking and wider channels due to reversible tautomerizations.^36^ These structural features facilitate enhanced electrolyte diffusion, more effective access to active sites, and improved ion-transport kinetics.^37^ Conversely, the irreversible enol-to-keto linkage in COP synthesis often results in kinetically trapped, irregular pore architectures, contributing to their amorphous nature and lower surface area. This was further corroborated by XRD (Figure 3c) analysis, where COFs exhibited sharp and intense diffraction peaks indicative of long-range order and higher surface area, whereas COPs showed broad, less-defined peaks due to disordered packing and heterogeneous pore-size distributions dominated by mesopores. Despite the denser structure, COPs may offer advantages in ion selectivity due to their smaller, more restricted pore environments. However, this often comes at the cost of reduced ion-transport efficiency, and the structural disparity between COFs and COPs presents a complementary relationship that can be leveraged to balance ion transport and selectivity in membrane applications, particularly in VRFBs, where such features are critical.^38^ The sulfonic segment within the covalent organic networks also finely promotes pore architecture while postering electrostatic interactions in the PBI composite membrane formation,^39^ further tuning the pore structure, and facilitating selective ion transport. This configuration effectively mitigates vanadium ion crossover, a crucial factor for improving the Coulombic efficiency and extending the cycle life in VRFBs in PBI composite membranes.

The surface and cross-sectional morphologies of the PBI/COF and PBI/COP composite membranes were analyzed using SEM, as shown in Figure 4a–f, along with the digital photograph in Figure S5. The SEM images confirm a dense and uniform structure across the entire membrane with no visible holes, pinholes, or cracks on the surface or cross sections of the composite membranes. The uniform dispersion of porous covalent organic networks within the PBI membrane enhances vanadium ion blocking through size-sieving ion selectivity, which is attributed to its consistently dense thickness (∼40 μm). This structure also mitigates electrolyte crossover between the anode and cathode.^40^ The dense and defect-free PBI layer and its composite membranes serve as benchmarks for evaluating the performance of the composite bilayer design. In this configuration, the dense PBI layer acts as an ion-selective barrier, significantly contributing to the overall membrane resistance. Thus, the sulfonic acid-bearing porous layers create improved ion-selective channels.

**Figure 4:**
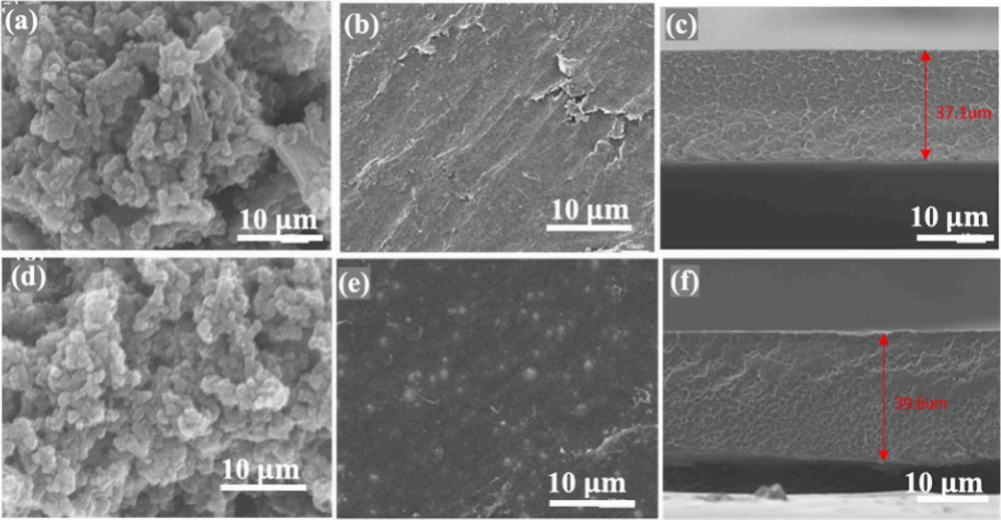
SEM images of (a) COF, (b) PBI/COF composite, (d) COP, (e) PBI/COP composite, and (c and f) their corresponding cross sections.

### 3.3 Physicochemical Properties

*W*_U_ and *S*_R_ are critical metrics for evaluating ion-exchange membranes under the standard conditions. As presented in Figure 5a and Table S2, the *W*_U_ values of PBI, Nafion, PBI/COP, and PBI/sCOF membranes range from 9.8 ± 0.12 to 29.2 ± 0.81, depending on their porosity and composition. The superior *W*_U_ observed in PBI/sCOF is attributed to its high density of hydrophilic functional groups, extensive hydrogen bonding, and electrostatic interactions between imidazole moieties and the porous organic networks compared with the protonated PBI membranes. The swelling ratio is another crucial parameter in VRFB membranes, influences electrolyte retention, ion transport, and structural integrity.^13^ The *S*_R_ of the membranes varies from 3.7 ± 0.19 to 16.31 ± 0.22 for Nafion, PBI, PBI/COP, and PBI/sCOF, respectively. The comparable *W*_U_ and *S*_R_ values of PBI/COP and PBI/sCOF can be attributed to their porous architectures and phase-separated morphology. Additionally, the incorporation of sulfonic acid networks in these composites enhances *W*_U_ while maintaining dimensional stability through continuous bonding interactions. The increased crystallinity further facilitates water adhesion to the porous structure, contributing to improved hydration and ionic conductivity.^41^ The interaction between sulfonic acid and imine groups in PBI/sCOF and PBI/COP plays a crucial role in optimizing the hydration properties while mitigating excessive ion dissolution. The introduction of sulfonic acid-functionalized COF and COP into PBI enables the composite membranes to achieve hydration levels comparable to Nafion while preserving structural integrity. The presence of sulfonic acid groups and their interactions within the porous organic network significantly enhance the IECs. This effect is evident in the IEC values shown in Figure 6f and Table S2, where PBI/sCOF exhibits the highest IEC due to its high density of SO_3_H groups, while the protonated PBI shows a lower IEC due to its limited number of ion-exchange sites, as determined by back-titration. The IEC of the membranes directly influences their hydration capacity and ion-transport properties.^42^ The enhancement of IEC, attributed to the formation of efficient ionic channels through continuous hydrogen bonding and electrostatic interactions. Another key metric for evaluating the membrane suitability in VRFBs is thermal stability. Promising thermal stability is a key metric for evaluating membranes used in VRFBs. The thermal stability of PBI composite membranes was assessed using TGA under a nitrogen (N_2_) atmosphere (Figure 5c). The initial weight loss of the PBI composite membranes occurred in two stages. The first stage, below 150 °C, corresponds to the evaporation of residual solvents and water molecules and over 500 °C, is related to the degradation of the polymer backbone and interconnected organic networks.^43^ Notably, the rigid and thermally stable structures of the COFs and COPs reinforce the PBI matrix, preventing thermal degradation at elevated temperatures. This integration ensures that the composite membranes maintain their structural integrity under high thermal stress, improving their stability and reliability in demanding electrochemical applications. For COF- and COP-integrated membranes, weight loss observed between 350 and 430 °C which attributed to the cleavage of −SO_3_H groups and imidazole units, which are thermally less stable functional groups. The final degradation, observed above 500 °C, corresponds to the breakdown of the polymer backbone itself. Importantly, PBI/COF and PBI/COP composite membranes demonstrate sufficient thermal stability for VRFB applications, which typically operate below 50 °C.

**Figure 5:**
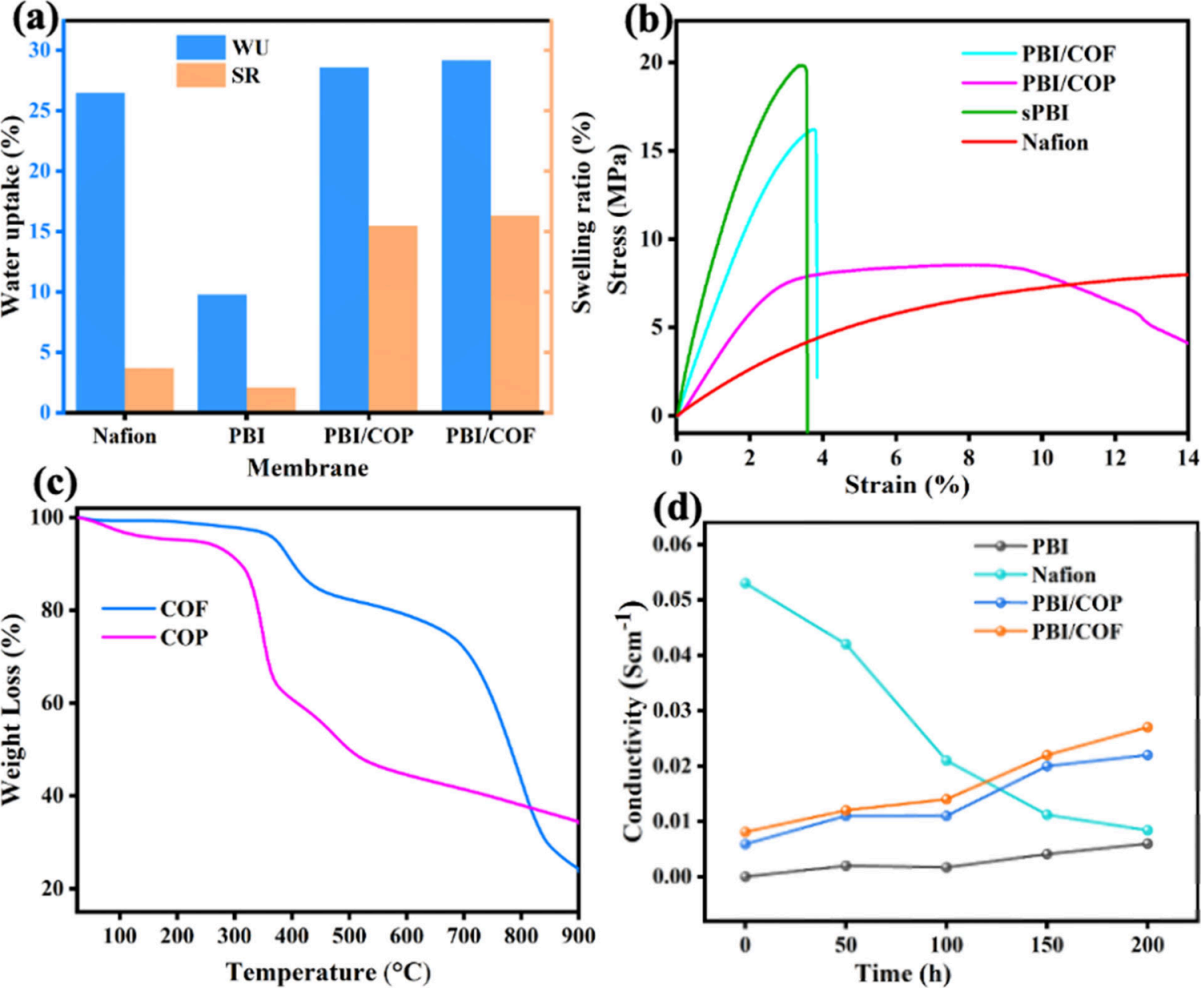
(a) Water uptake and swelling ratio, (b) mechanical properties, (c) thermal properties of COF and COP, and (d) acidic stability of the membranes over time.

**Figure 6:**
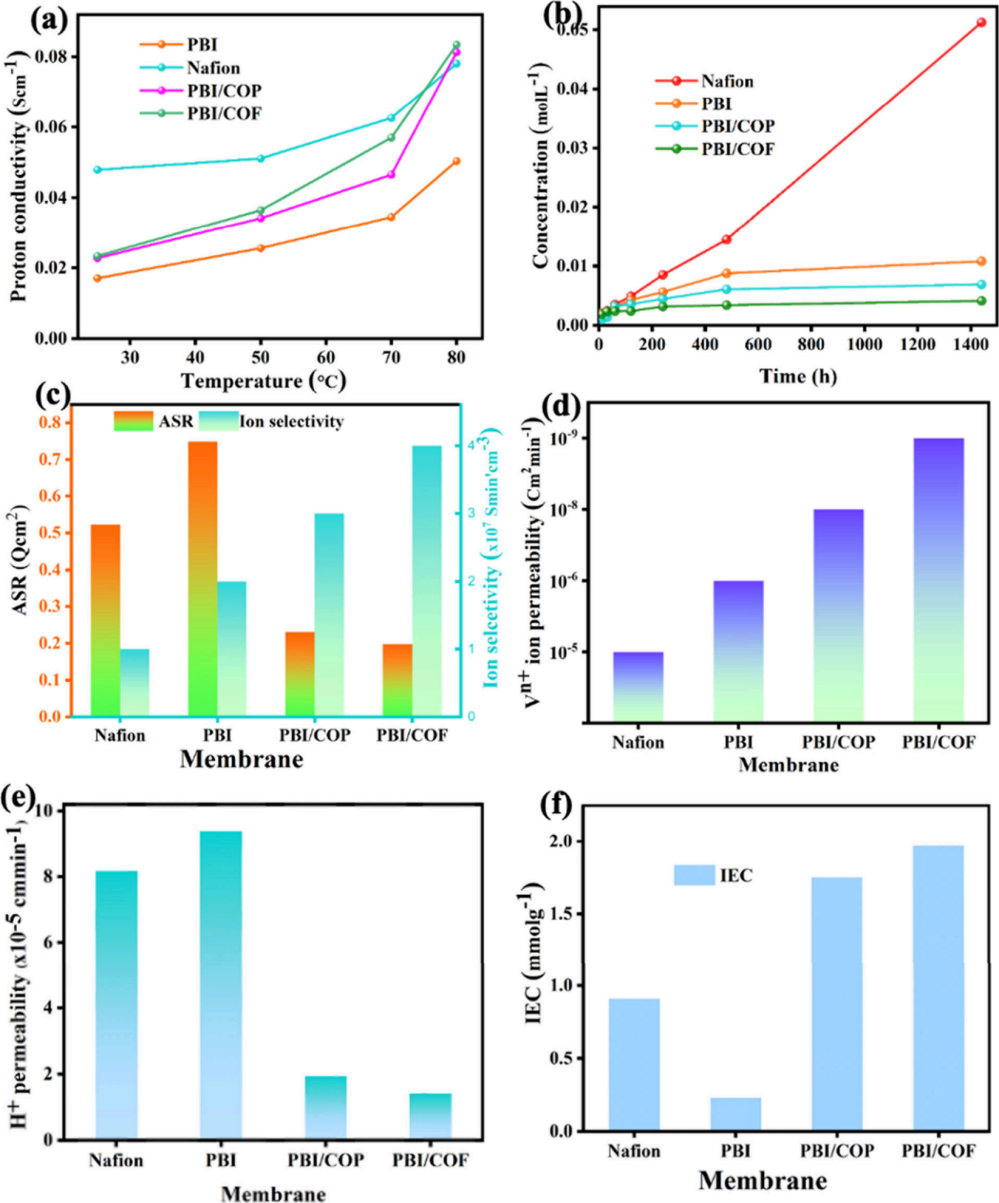
(a) Proton conductivity, (b) concentration changes of VO^2+^ ions over time, (c) ASR and ion selectivity, (d) V^*n*+^ ion permeability, (e) proton permeability, and (f) IEC of the membranes.

Furthermore, the mechanical properties of PBI/COF, PBI/COP, and recast Nafion membranes were tested at 25 °C to assess their suitability and the effects of covalent organic network composites, as shown in Figure 5b. The mechanical robustness of membranes is crucial for ensuring their structural integrity under demanding operational conditions, thereby enhancing battery durability in long-term applications. The mechanical strength of a membrane is influenced not only by the inherent properties of the base polymer but also by the integrated amount and type of composite materials. Pure PBI exhibits excellent tensile strength and regains its original shape upon deformity, which is comparable to that of Nafion. In composite membranes, the incorporation of covalent organic networks often enhances mechanical strength by controlling void spaces within the base polymer matrix. Specifically, the integration of sulfonic acid-containing covalent organic networks, derived from TFP and DABA, into the PBI base polymer improves the overall membrane properties while preserving the intrinsic internal structure of PBI.^44^ Among these composites, PBI/COF shows the highest tensile strength of 19.56 and 14.92 MPa of PBI/COP. This is reflected in its elongation at break, measured at 15.54%. This enhancement is attributed to the in situ growth of COFs within the PBI matrix and the strong electrostatic interactions between imidazole groups and the sulfonic acid ends of the organic network. These factors collectively enhance the tensile strength and overall properties of the composite membranes, ensuring membranes withstand swelling, mechanical stress, and cycling, enhancing durability, safety, and efficiency while maintaining ion selectivity and ease of handling.^45^

Chemical stability is a critical factor in the performance and longevity of VRFB applications. The acidic environment stability of PBI, Nafion, PBI/COP, and PBI/COF composite membranes was assessed through the evaluations of proton conductivity followed by an ex-situ oxidative stability test. As revealed in Figure 5d, the ex situ chemical stability of the membranes was evaluated by immersing membranes of specified geometric size in a 0.1 M VO^2+^/VO_2_^+^ solution containing 3 M H_2_SO_4_ at 30 °C for 200 h. No physical alterations, such as cracks, pinholes, or surface interruptions, were observed, attributed to the robust material composition, uniform dense structure, and chemical compatibility of PBI with the functionalized sulfonic acid layers of TFP and DABA combinations. Moreover, the conductivity of the membranes was assessed to determine the effects of the acidic treatment. These assessments confirmed that the membranes’ conductivity remained stable over time for the prepared composite membranes due to the facile integration of PBI with composites, along with π–π stacking, hydrogen bonding, and strong electrostatic interactions, enhancing their chemical and structural durability under prolonged acidic vanadium electrolyte. No significant variations in conductivity were observed as compared with its proton conductivity in Figure 6a, highlighting the membranes’ robust structural and chemical integrity under oxidative conditions. However, the conductivity of Nafion gradually declines, which can be attributed to the oxidative degradation of sulfonic acid groups (−SO_3_H) in the presence of mild oxidizing agents like VO^n+^, reducing its IEC and impairing hydration, thereby limiting water retention essential for efficient proton conductivity. Furthermore, negligible changes in VO^2+^/VO_2_^+^ concentrations were detected even after 14 days of immersion, underscoring the exceptional oxidative stability of the composite membranes.^46^ This impressive chemical stability establishes PBI-based composite membranes as durable candidates for VRFBs, capable of withstanding prolonged exposure to harsh acidic and oxidative environments without compromising performance. Their stability and durability outperform conventional materials such as Nafion, ensuring reliability and longevity under demanding VRFB operational conditions.

### 3.4 Ion Transport, Selectivity, and Area-Specific Resistance (ASR)

Proton conductivity is one of the leading factors in VRFB efficiencies, governed by the transport of H^+^ and related ions within the membranes.^47^ As shown in Figure 6a, the proton conductivity of PBI, the composites, and Nafion membranes exponentially increases with the scale of temperature and the IEC values and runs from 0.0171 S cm^–1^ of PBI to 0.0234 S cm^–1^ of PBI/COF at 30 °C; the conductivity of PBI composites slightly surpasses the conductivity of Nafion as the rise of temperature due to strong hydrogen-bonding and electrostatic interactions between imidazole and sulfonated COFs, enhancing ionic mobility, aggregation, and microphase separation to facilitate efficient ion transport. The pristine PBI matrix exhibits limited proton conductivity; the integration of COFs and COPs significantly enhances their proton conductivity and selectivity. This improvement stems from a size-selective transport mechanism that distinguishes smaller hydrated protons from larger vanadium ions. Hydrated protons, being smaller than the pore size of the PBI/COF–COP composite membrane, traverse the channels with ease, whereas vanadium ions are effectively blocked. In an acidic environment, protonated imine groups in PBI, activated by sulfonic acid components, facilitate proton transfer through the robust hydrogen-bonding network shown in Scheme 2. This network enables proton hopping via the Grotthuss mechanism.^48^ The Donnan exclusion effect of the positively charged imine groups further contributes to vanadium ion blocking, ensuring high proton selectivity and proton permeation. The proton permeability of the VRFB membrane was tested by using a diffusion cell following the vanadium ion permeability protocol. While proton conductivity enables efficient ionic transport, controlled proton permeability is crucial for minimizing electrolyte crossover.^49^ As shown in Figure 6b, PBI and its composites achieve a balance, with proton permeability ranging from 8.1 × 10^–5^ to 1.41 × 10^–5^ cm^2^ min^–1^, slightly lower than that of Nafion. This balance enhances Coulombic efficiency and capacity retention by reducing crossover while maintaining effective ionic transport. The relative ratio of ionic conductivity to vanadium ion permeability is a critical parameter for evaluating ion selectivity in VRFBs as a function of the area resistance. As illustrated in Figure 6c, the PBI/COP and PBI/COF composite membranes demonstrated the highest ion selectivity, measured at 2.412 × 10^7^ and 2.529 × 10^8^ S min cm^–3^, which significantly exceeds that of Nafion (2.10 × 10^6^ S min cm^–3^). This superior selectivity indicates that the composite membrane more effectively facilitates the transport of protons while suppressing the crossover of VO^*n*+^ ions.^50^ Thus, the PBI/COP-COF composite membrane not only exhibited the highest ion selectivity but also demonstrated low ASR (Figure 6c), reflecting its remarkable proton conductivity, an essential attribute for efficient energy transfer in VRFBs. The PBI/COF composite membrane exhibited a lower ASR of 0.198 Q cm^2^ compared to Nafion (∼0.5 Q cm^2^) due to improved ionic channels.

**Scheme 2:**
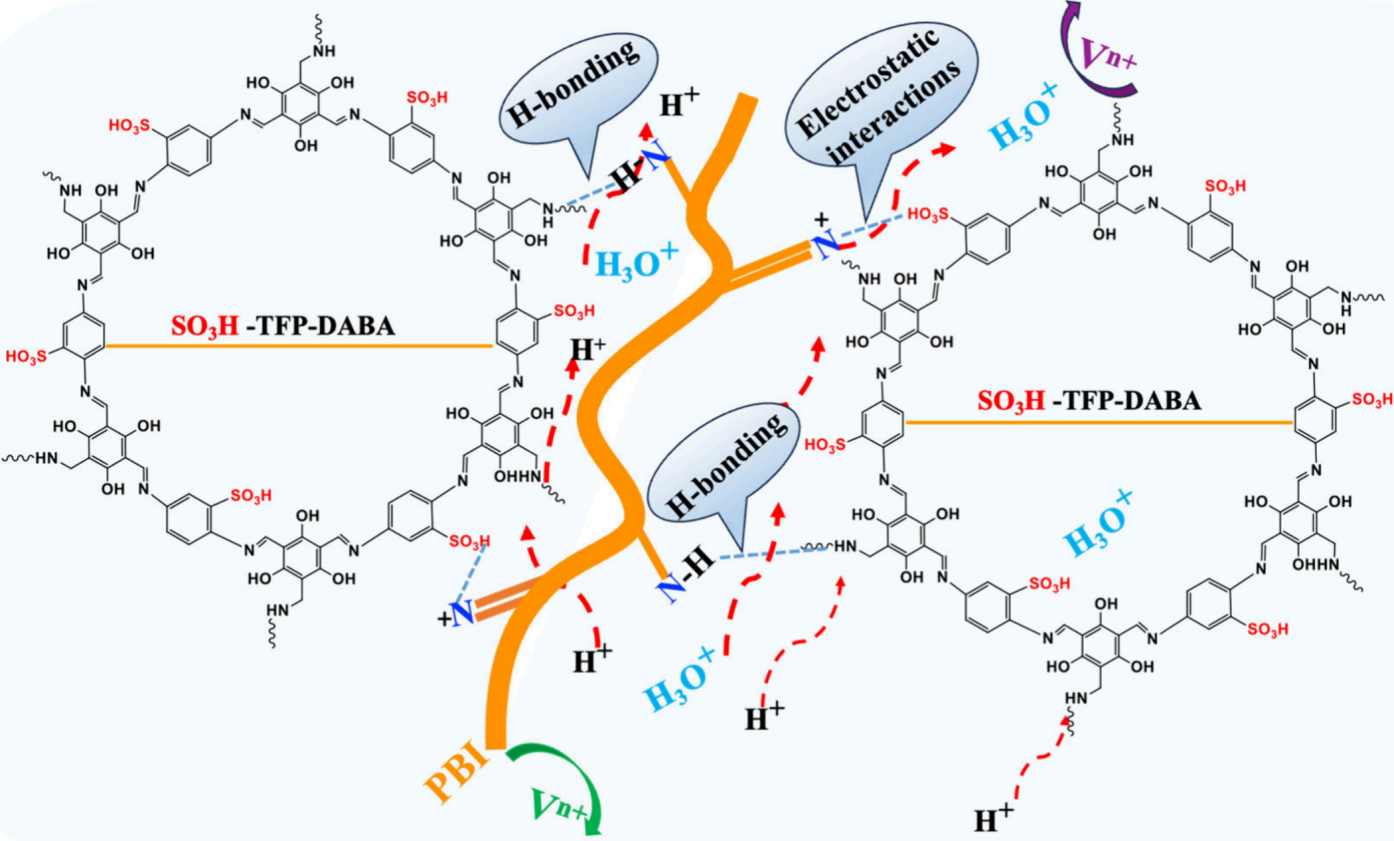
Proton-Transport Pathway

Furthermore, high vanadium ion permeability in PEMs can lead to low efficiency and rapid capacity decay, limiting the practical application of VRFB systems.^51^ To compare the vanadium ion permeabilities of PBI and composite membranes with Nafion, diffusion tests were conducted. As shown in Figure 6d, the permeability of VO_2_^+^ through PBI, PBI/COF, and PBI/COF membranes is significantly lower than that of Nafion. The permeability was determined by linearly fitting the concentration versus diffusion time (Figure 6b). Nafion exhibits a permeability of 1.92 × 10^–5^ cm^2^ min^–1^, 1 or 2 orders of magnitude higher than the PBI, PBI/COF, and PBI/COF membranes. This indicates that these membranes are more effective at preventing vanadium ion crossover and improving Coulombic efficiency and capacity retention. For PBI, PBI/COF, and PBI/COF membranes, vanadium permeability ranges approximately from 1 × 10^–6^ to 1 × 10^–9^ cm^2^ min^–1^, depending on the crystallinity and sieving capability of the membranes. The enhanced ion-blocking capability is attributed to the continuous sieving and interconnected interactions between the base polymer and the porous materials in regular stacking.

### 3.5 Membrane Performance in VRFBs

The single-cell battery performance and cycling stability of PBI composite membranes and commercially available Nafion membranes were evaluated at current densities ranging from 40 to 100 mA cm^–2^, as presented in Figure 7a–d. At a low current density of 40 mA cm^–2^, the PBI/COF membrane achieves Coulombic efficiencies ranging from 94.18 to 99.19%, surpassing those of Nafion (93.04–96.74%), followed by PBI/COP (94.18–98.46%). This outcome underscores the superior vanadium-ion-blocking capability of the PBI composite membranes compared to Nafion, whereas the VE of the PBI/COF-based performance runs from 75.99 to 93.56% at a current density between 40 and 100 mA cm^–2^, showing a substantial improvement compared to Nafion (68.11–90.18%), demonstrating its ability to reconcile proton permeability and selectivity for enhanced performance. Notably, the membrane sustains an ultrahigh CE (99.19%) while maintaining this high VE, effectively overcoming the traditional trade-off in membrane design for acid-supporting VRFBs.

**Figure 7:**
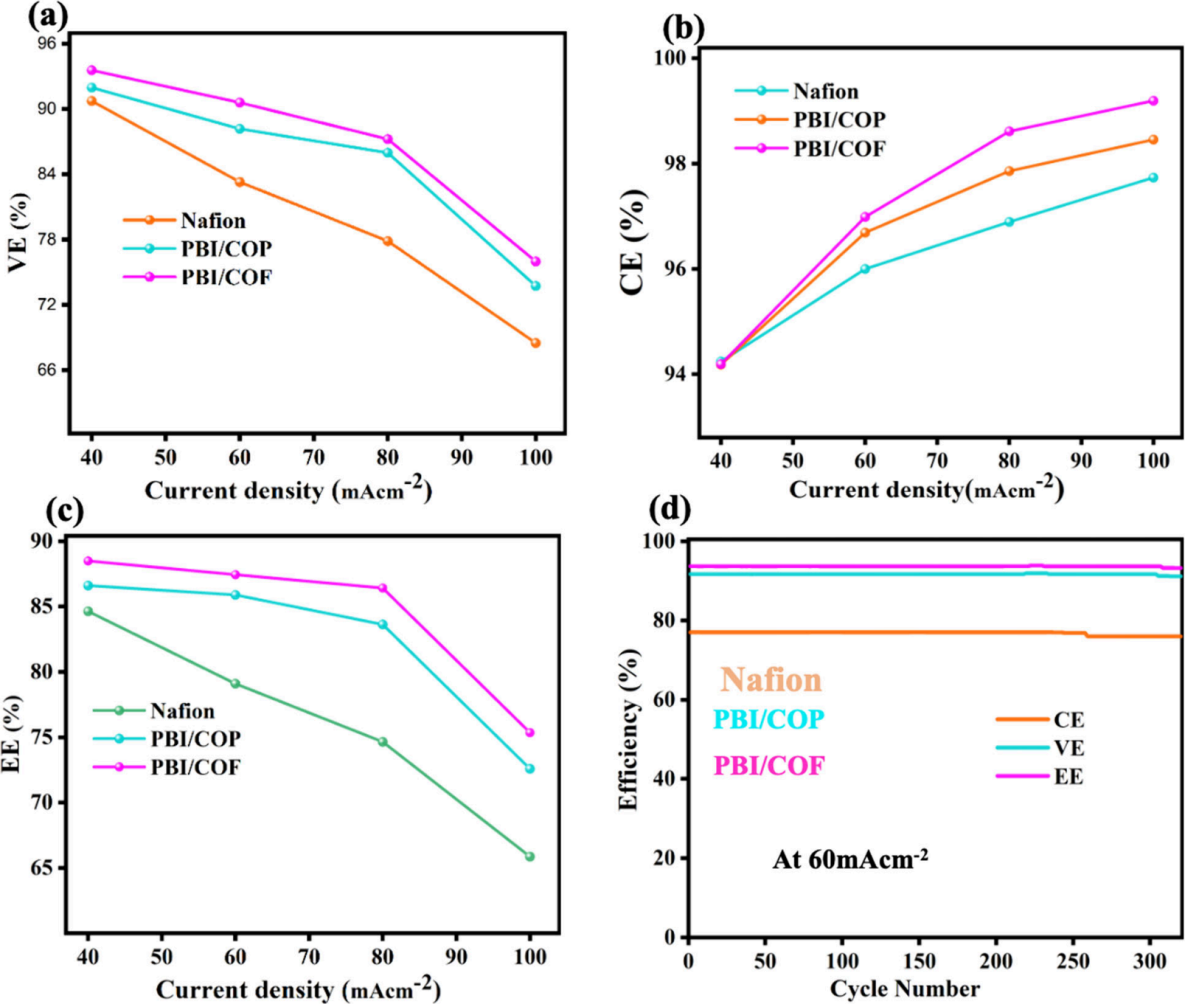
(a–c) VRFB performance metrics (CE, VE, and EE) and (d) long cycle stability for Nafion and PBI composite membranes.

The EE (88.5%), the most comprehensive indicator of the VRFB performance, underscores the superiority of the PBI/COF membrane. With an EE of 88.5%, it markedly outperforms pristine Nafion (84.65%), which is attributed to its optimized pore architecture, which ensures precise proton/vanadium ion sieving. Across a current density range of 40–100 mA cm^–2^, the PBI/COF membrane maintains an EE range of 88.5–75.37%, significantly surpassing that of Nafion, which ranges from 84.65% to 65.88%. At higher current densities, shorter vanadium ion crossover times improve the CE, although the VE declines due to increased ohmic polarization losses.^52^ At lower current densities, the PBI/COF membrane demonstrates dominance, driven by its exceptional VO^2+^ blocking capability and enhanced CE. These results collectively confirm the transformative potential of sulfonated organic networks in PBI composites for optimizing the membrane performance in next-generation VRFBs. As presented in Figure 7a,c, PBI composite membranes exhibit remarkable Coulombic efficiency, voltage efficiency, and energy efficiency, outperforming Nafion membranes in VRFB single cells. This superior performance is attributed to their exceptional capability to suppress vanadium ion crossover. The integration of porous covalent organic nanopolymers functionalized with sulfonic acid groups into the PBI matrix significantly minimizes vanadium ion permeability, further augmenting their efficacy. Although the voltage efficiency shows a declining trend at higher current densities due to increased internal resistance, PBI composite membranes consistently outperform Nafion, particularly at elevated current densities (e.g., 100 mA cm^–2^). Moreover, as shown in Table 1, the EE versus current density of these membranes surpasses those reported in related studies, highlighting their potential to enhance high-power-density VRFBs by achieving an optimal balance between ionic selectivity and proton conductivity.

**Table 1:** Comparison of the EE Performances of Sulfonic Acid-Containing COF/COP Composite PBI Membranes with Related Studies at Current Densities of 40–60 mA cm^–2^

membrane	current density (mA cm^–2^)	EE %	reference
50PS–PBP-30	60	81.7	^6^
mcPBI-S-32%	60	77.55	^40^
SPEKS/sGO	40	82.5	^53^
PVA-SiO_2_-Sn	40	75.4	^54^
sPBI–MGE-2	60	83.81	^55^
[Nafion/(WO_3_)_0.587_]	50	65	^56^
S/NH_2_-GO-2	50	84.9	^57^
SP/TpTG-3	60	87.05	^46^
SPEEK/PPO-TTA-15	40	90.3	^58^
PBI/COP	60	86.09	this work
PBI/COF	60	88.00	this work

The long-term cycling stability and energy efficiency of the PBI composite and Nafion membranes were evaluated over 320 cycles, as shown in Figure 7d. The CE and VE remained stable across all membranes at 60 mA cm^–2^ in a single-cell VRFB stack. In contrast, the EE of the Nafion membrane gradually declined by 2.4% over time due to its initially higher proton conductivity under normal conditions. After 320 cycles, the average VE of the Nafion membrane decreased to 76.71%, approximately 2.4% lower than those of the PBI/COP and PBI/COF membranes, which remained structurally stable. Consequently, the EE of the PBI/COF and PBI/COP membranes reached 84.72% and 87.23%, respectively, surpassing that of Nafion across the tested current density range. These findings underscore the superior cycling stability and higher EE of PBI composite membranes, highlighting their potential as promising alternatives for long-term VRFB applications.

## 4 Conclusion

In this study, sulfonic acid-containing covalent organic frameworks (sCOFs) and polymers (sCOPs) were incorporated into porous PBI composite membranes via interfacial condensation reactions, considering solvent effects. The sulfonic acid groups effectively protonate PBI’s imidazole rings, enhancing proton conductivity and transport. Additionally, they mitigate vanadium ion crossover through size sieving and electrostatic repulsion, optimizing the membrane’s porosity and structural integrity for improved VRFB performance. The thickness of the dense layer was precisely controlled by adjusting the PBI/COP-COF composite content in the casting solution, enabling fine-tuning of the proton conductivity, vanadium ion permeability, and overall ion selectivity. The PBI/COP-COF composite membranes exhibited low VO^2+^ permeability (2.24 × 10^–8^ cm^2^ min^–1^), significantly lower than those of Nafion (8.1 × 10^–8^ cm^2^ min^–1^) and pristine PBI (9.23 × 10^–8^ cm^2^ min^–1^) membranes, along with high proton conductivity (0.023 S cm^–1^) at normal conditions. The single-cell performance further demonstrated a Coulombic efficiency (CE) of 99.19%, a voltage efficiency (VE) of 93.56% at 40 mA cm^–2^, and an energy efficiency (EE) of 88.5% at 40 mA cm^–2^, surpassing those of pristine PBI, Nafion, and related reports with the stated current density. These features, combined with low-cost, position PBI covalent organic network composite membranes as viable alternatives to Nafion for efficient, durable, and cost-effective VRFB systems, and the study establishes a solid foundation for developing PBI covalent organic network composite proton-selective membranes with tailored pore structures and chemical functionalities. These advancements hold significant promise for diverse electrochemical applications and sustainable energy storage technologies, paving the way for further optimizations.

## Supplementary Material



## Data Availability

All data are included in the manuscript, with additional details available upon request.

## Notes

Synthesis routes of COF and COP (Scheme SI), energy-dispersive X-ray spectroscopy of COF and COP (Figure S1), surface elemental composition of Nafion, PBI, and composite membranes (Table S1), BET analysis of PBI/COF and PBI/COP composite membranes (Figure S2), XRD crystallinity spectra of COF and COP (Figure S3), physicochemical properties of Nafion, PBI, and composite membranes (Table S2), and optical photographs of membranes with dispersed casting solutions (Figure S4) (PDF)
