# Supplementary material for: Polybenzimidazole Membranes Doped with Sulfonic Acid-Containing Covalent Organic Frameworks and Polymers for Enhanced Performance in Vanadium Redox Flow Batteries
Source: ACS Appl Mater Interfaces. 2025 Jun 23;17(26):37826–39. doi: 10.1021/acsami.5c00568 (PMC12232264; doi:10.1021/acsami.5c00568)
Supplement: Supplementary file 1 [file am5c00568_si_001.pdf]

Supporting Information

Polybenzimidazole Membranes doped with Sulfonic Acid-Containing Covalent Organic Frameworks and Polymers for Enhanced Performance in Vanadium Redox Flow Batteries

Beyadgalem Endawoke Anley[a], Cheng-Ju Yu[a], Tsung-Yun Wu[a], Chun-Chiang Huang[c], Jun-Sheng Wang[c], Hsieh-Chih Tsai[a, b, d*]

a. Graduate Institutes of Applied science and Technology, National Taiwan University of science and Technology, Taipei 106, Taiwan, ROC.
b. Advanced Membrane materials center, National Taiwan University of science and Technology, Taipei 106, Taiwan
c. Taiwan Instrument Research Institute, National Applied Research Laboratories, Hsinchu 302, Taiwan.
d. R&D Center for Membrane Technology, Chung Yuan Christian University, Chungli, Taoyuan 320, Taiwan

Correspondence: h.c.tsai@mail.ntust.edu.tw (H.C.T), tel.: +886 -2-227303779 (H. C. T)

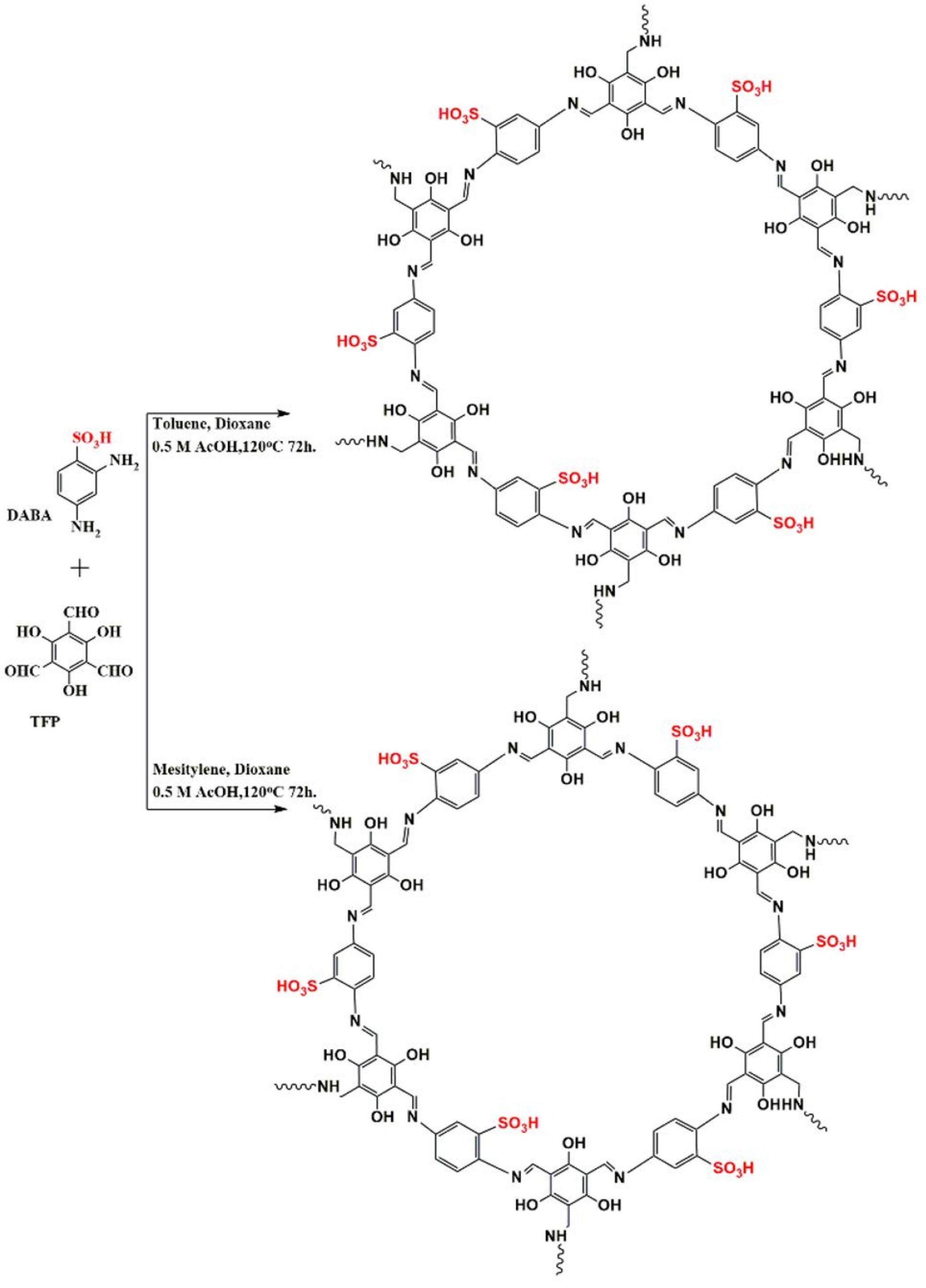

Scheme SI: Synthesis routes of COF and COP

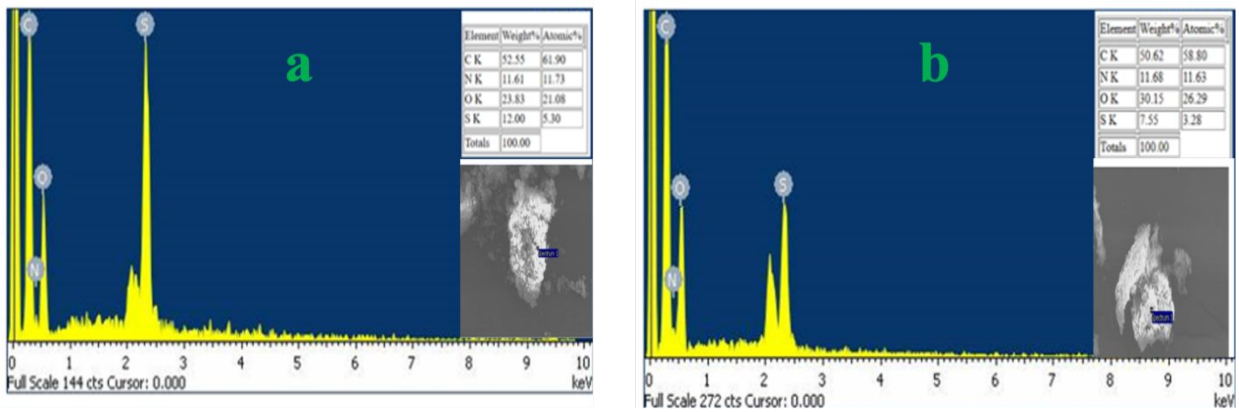

Figure S1:Energy dispersive X-ray spectroscopy (EDX) of COF (a) and COP (b)

Table S1: Surface Elemental Composition (EDS) of Nafion, PBI, and composite membranes

Membranes	Weight (%)				Atomic (%)			
Nafion	C	N	O	S	C	N	O	S
PBI	75.06	14.10	10.76	0.09	78.79	12.69	8.48	0.03
PBI/COP	62.59	11.52	24.85	1.03	68.39	10.80	20.39	0.42
PBI/COF	43.75	9.02	46.19	1.09	50.56	8.94	40.03	0.47

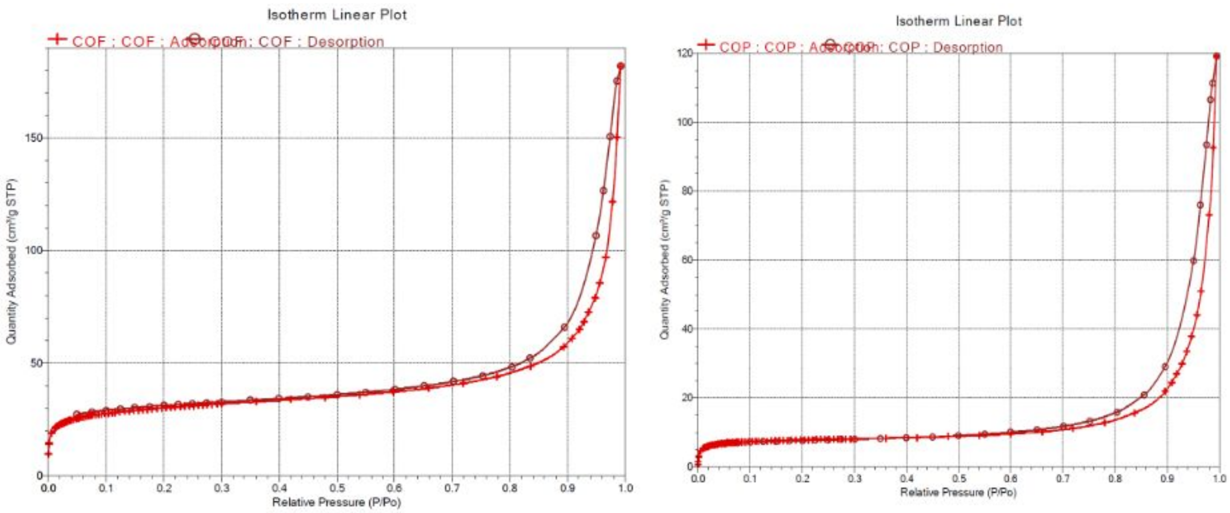

Figure S2:BET analysis of the PBI/COF, COP composite membranes

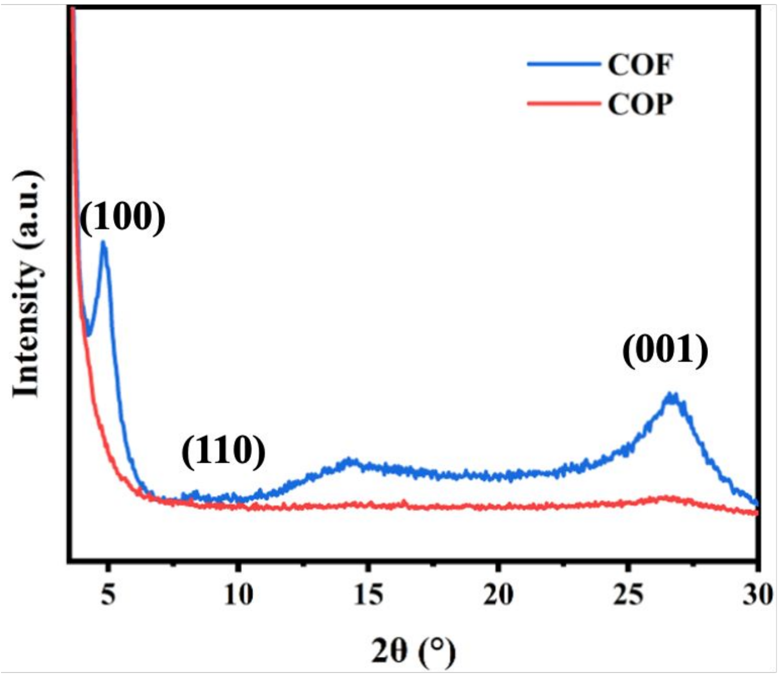

Figure S3:XRD crystallinity spectra pattern of COF and COP

X-ray diffraction (XRD) analysis was performed to investigate the crystallinity of the nanosheets formed at the interface between each phase. As shown in figure 3S, the XRD pattern exhibits a prominent crystallization peak at 4.7° for the COF, confirming the formation of well-ordered

crystalline structures. In contrast, the broad or nearly absent peaks for COP indicate its amorphous nature compared to COF. The peak at 4.7° corresponds to the (100) plane, signifying the orderly stacking of COF layers. Additionally, the slightly broader peak at higher 2θ angles likely reflects defects in the π-π stacking between consecutive COF layers.

Table S2:Physicochemical properties of Nafion, PBI, and Composite membranes

Membrane	W_U (%)	S_R (%)	IEC ($mmolg^{-1}$) [a]	TS (MPa) [b]	Elongation at Break (%) [c]
Nafion	26.5 ± 0.21	3.7 ± 0.19	0.9	8.18	7.23
PBI	9.8 ± 0.12	2.1 ± 0.13	0.23	8.74	7.41
PBI/COP	28.6 ± 2.01	15.5 ± 0.02	1.75	14.92	11.85
PBI/COF	29.2 ± 0.81	16.31 ± 0.22	1.96	19.56	15.54%.

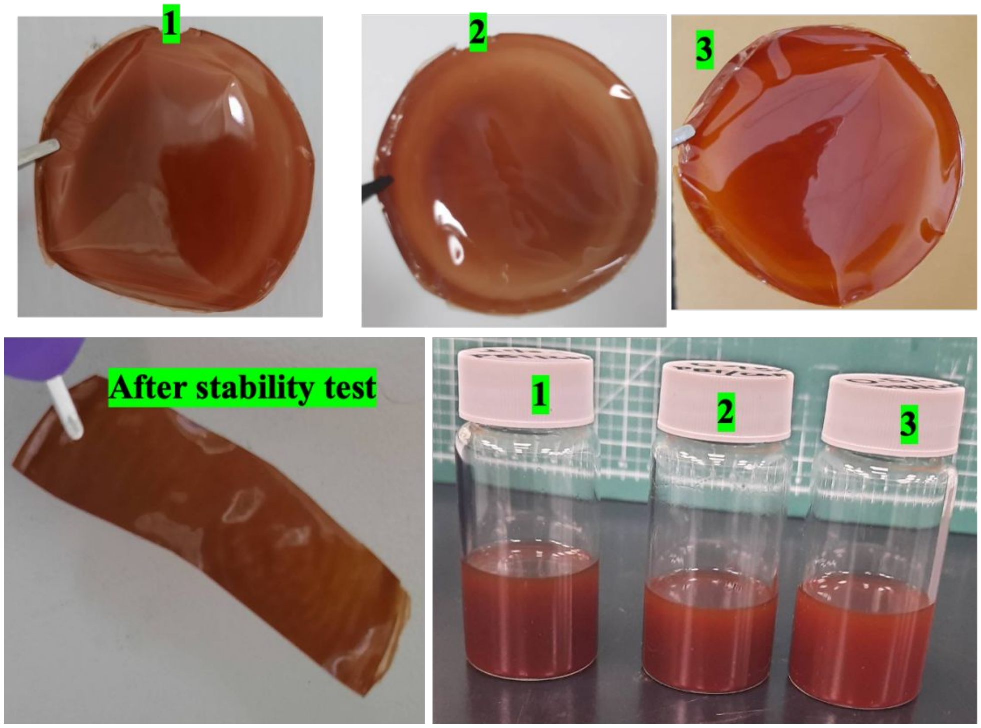

Figure S4: Optical photographs of the membranes with the dispersed casting solutions
